# Lack of PNPase activity in *Enterococcus faecalis* 14 increases the stability of EntDD14 bacteriocin transcripts

**DOI:** 10.1038/s41598-023-48619-y

**Published:** 2023-12-18

**Authors:** Rabia Ladjouzi, Anca Lucau-Danila, Paloma López, Djamel Drider

**Affiliations:** 1 UMR Transfrontalière BioEcoAgro INRAe 1158, Univ. Lille, INRAE, Univ. LiègeUPJVYNCREA, Univ. Artois, Univ. Littoral Côte d’OpaleICV-Institut Charles Viollette, 59000 Lille, France; 2 Department of Microorganisms and Plant Biotechnology, Biological Research Center-Margarita Salas (CIB-Margarita Salas, CSIC), Madrid, Spain; 3 Present Address: EA DYNAMYC 7380, Université Paris-Est Créteil (UPEC), École Nationale Vétérinaire d’Alfort (ENVA), USC Anses, 94000 Créteil, France

**Keywords:** Microbiology, Molecular biology

## Abstract

A mutant deficient in polynucleotide phosphorylase (PNPase) activity was previously constructed in *Enterococcus faecalis* 14; a strain producing a leaderless two-peptide enterocin DD14 (EntDD14). Here, we examined the impact of the absence of PNPase on the expression and synthesis of EntDD14, at the transcriptional and functional levels. As result, EntDD14 synthesis augmented in line with the growth curve, reaching a two- to fourfold increase in the *ΔpnpA* mutant compared to the *E. faecalis* 14 wild-type strain (WT). EntDD14 synthesis has reached its highest level after 9 h of growth in both strains. Notably, high expression level of the *ddABCDEFGHIJ* cluster was registered in *ΔpnpA* mutant. Transcriptional and in silico analyses support the existence of *ddAB* and *ddCDEFGHIJ* independent transcripts, and analysis of the fate of *ddAB* and *ddCDEFGHIJ* mRNAs indicated that the differences in mRNA levels and the high EntDD14 activity are likely due to a better stability of the two transcripts in the *ΔpnpA* mutant, which should result in a higher translation efficiency of the *ddAB* EntDD14 structural genes and their other protein determinants. Consequently, this study shows a potential link between the mRNA stability and EntDD14 synthesis, secretion and immunity in a genetic background lacking PNPase.

## Introduction

In nature, microorganisms use various methods for intercommunication and defense. One of these consists of production and release of bacteriocins, which are a group of multifunctional ribosomally synthesized peptides/proteins produced by all major lineages of bacteria and archaea^1–3^. Bacteriocins are abundant, and more than 99% of bacteria are thought to produce at least one bacteriocin^4^. Nevertheless, bacteriocins produced by Gram-positive bacteria, mainly those produced by lactic acid bacteria (LAB), are of particular interest due to their industrial use (e.g. nisin) and that many of their producing bacteria have the status of generally recognized as safe (GRAS) microorganisms. To date hundreds of bacteriocins have been described, and different classifications were suggested to compile these molecules based on their primary structure, amino-acids sequences, mode of action^5–9^. Nonetheless, these classifications undergo constant modifications as new findings appear. Bacteriocins can display either a narrow or broad spectrum of activity^7^. Producing strains protect themselves from lethal action of their own bacteriocins by using immunity proteins^10^, which are encoded by genes usually located in the same operon as the genes encoding the structural genes of bacteriocins^6^. The interaction of bacteriocins with the target cell is thought to occur in two distinct steps. The first corresponds to the adsorption in the cell surface via specific receptors. This interaction is likely reversible and desorption of the bacteriocin leaves the target cell intact. The second step irreversibly leads to cell damage and death^11^. Research on bacteriocins is growing because of their potential clinical applications, as resistance to conventional antibiotics has become a concern^12^. In this context it was reported that deaths associated with antibiotic resistance is a serious public health problem around the world. According to the World Health Organization^13^, the number of deaths yearly registered worldwide due to antibiotic resistance is about 700,000. In Europe, this number arose from 25,000 to 33,000 from 2015 to 2017^14^, delineating a global health emergency. Bacteriocins are among the suggested alternatives to antibiotics for effective control of drug-resistant pathogens^15^. The production of bacteriocins from a single strain fermentation is laborious and time-consuming with often a low yield^16^. Several studies were conducted with the aim to improve these yields. These consist of co-cultures between the producing strain and other strains triggering genetic expression synchronization and production of bacteriocins^17,18^. The presence of additional bacteria in co-culture acts as a stress signal, and usually enhances the production of bacteriocin. Similarly, several studies were conducted at the molecular level to express the native or synthetic genes encoding bacteriocins in heterologous hosts, mostly in *E. coli*^19^.

In terms of regulation of leaderless bacteriocins, some studies have reported the role of transcriptional regulators^20,21^ and the environmental factors such as nutrition-adaptation or temperature variation in controlling bacteriocin biosynthesis^22,23^. Here, we provide new insights for controlling the expression of genes encoding bacteriocins. Indeed, we correlate the expression of genes *ddA* and *ddB* coding for EntDD14 in *E. faecalis* 14^24^ to ribonucleases machinery, and particularly to PNPase, which is a major and pleiotropic 3′–5′ exoribonuclease^25^. This study establishes that the absence of PNPase activity leads to a higher stability of transcripts of the genes required to synthesize and generate an active extracellular EntDD14 coupled to a higher antibacterial activity. To the best of our knowledge, this is the first report showing the role of a major ribonuclease in the expression and production of bacteriocin, namely EntDD14, paving the way to a novel approach to control production of bacteriocins.

## Results

### The *E*. *faecalis *Δ*pnpA* mutant deficient in PNPase activity has more antibacterial activity than the *E*. *faecalis* 14 wild-type strain

**Figure 1:**
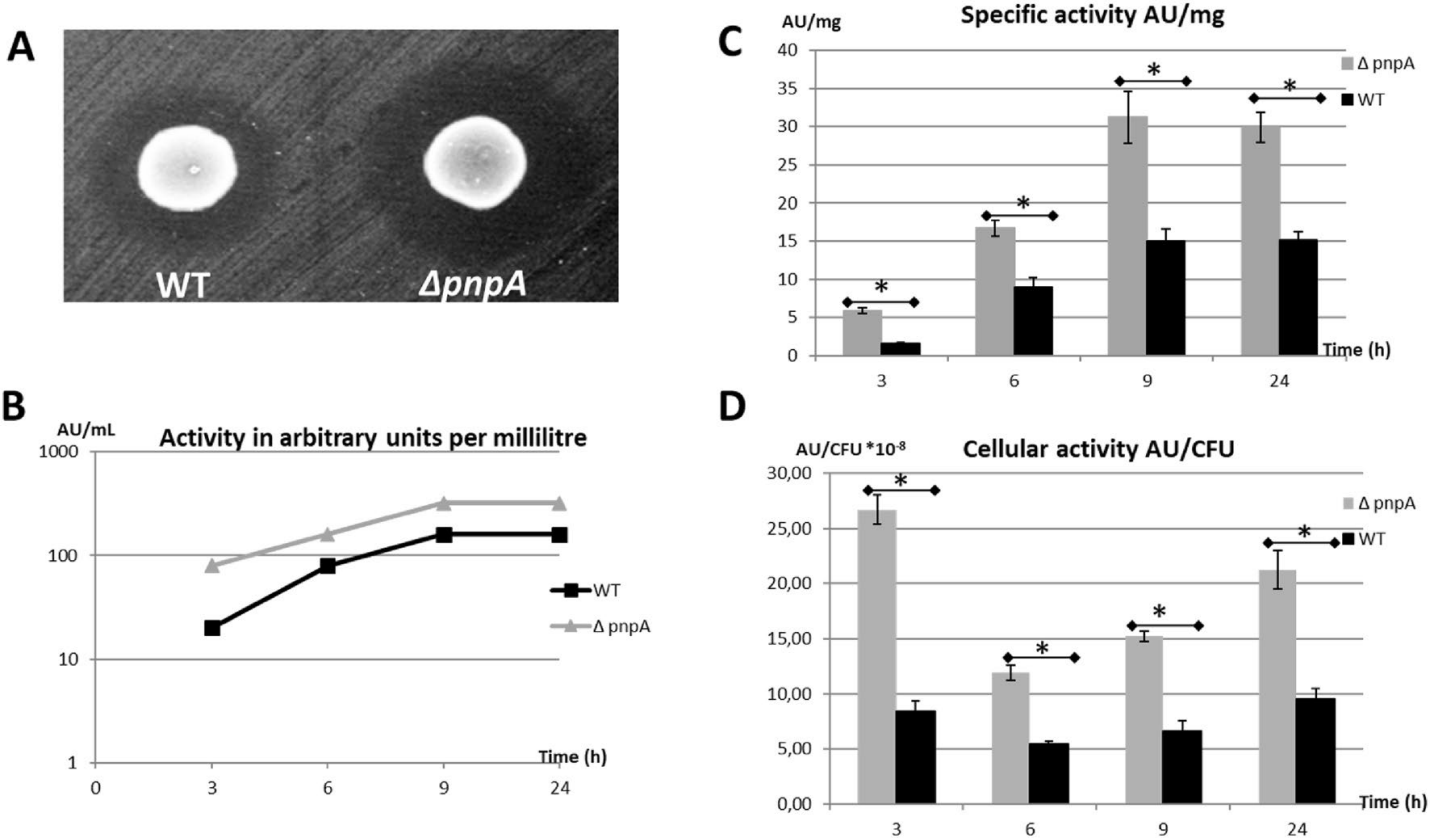
Anti*-Listeria innocua* activity of *E. faecalis* 14 and *∆pnpA* strains. (**A**) Anti-*L. innocua* using spot test. The zone of inhibition indicates the susceptibility of the bacterial lawn (*L. innocua* ATCC33090) to the produced EntDD14; (**B**) Anti-*L. innocua* activity using the well diffusion test quantified by AU/mL; (**C**) Specific activity of the cell-free supernatants expressed in AU/mg; (**D**) Anti-*L. innocua* cellular activity expressed in AU/CFU. The vertical bars represent the standard deviations and the asterisks are used when the *P* value is significant (*P* < 0.05). The data are the means of three independent experiments.

*L. innocua* ATCC33090 was used to assess the antibacterial activity of the EntDD14, which was previously reported to be the unique anti-*Listeria* activity compound present in the cell-free supernatant (CFS) of the WT strain^26^. Using the spot-on-lawn method, cultures (4 µL) of the WT or *∆pnpA* mutant were deposited on BHI plates previously inoculated with *L. innocua* ATCC 33090. After 24 h of incubation under appropriate conditions, the *∆pnpA* mutant exhibited a halo around the spot larger than that measured for the WT, indicating a possible bacteriocin accumulation (Fig. 1A). Next, the anti-*Listeria* activity was determined by the well-diffusion method using CFS issued from the WT and *∆pnpA* mutant. Data from these experiments showed that the antibacterial activity of the CFS from the *∆pnpA* mutant was significantly (P < 0.05) higher than that of the WT (Fig. 1B). In fact, after 3 h of growth, the ∆*pnpA* mutant displayed a fourfold higher anti-*L. innocua* activity than the WT (80 AU/mL versus 20 AU/mL), indicating an earlier production stage in the *∆pnpA* strain. However, after 6 h and 9 h of growth, anti-*Listeria* activity was two-fold higher in the *∆pnpA* mutant strain than in the WT strain. Thus, a total activity of 160 and 320 AU/mL were registered for *∆pnpA* mutant versus a total activity of 80 and 160 AU/mL for the WT after 6 h and 9 h, respectively. Nonetheless, after 24 h of growth, the anti-*Listeria* activity remained unchanged for both strains. The levels of EntDD14 specific activity referred to mg determined with the CFS of either strain correlated with the high anti-*Listeria* activity observed in the *∆pnpA* mutant (Fig. 1C). In fact, after 3 h of growth, the specific activity of the bacteriocin produced by the *∆pnpA* mutant was 3.64-fold higher than that of the WT (*P* = 0.002), (5.93 ± 0.35 AU/mg versus 1.626 ± 0.056 AU/mg). Moreover, after 6 h, 9 h and 24 h of growth, the *∆pnpA* mutant still showed a specific activity 1.85, 2.08 and 1.98-fold higher than the WT, with *P* values of 0.002, 0.004 and 0.008, respectively (Fig. 1C). The anti-*Listeria* activity (Fig. 1B), and the specific activity of the supernatants (Fig. 1C) reached their highest values after 9 h of growth, when both strains reached the beginning of the stationary phase, and before this phase was stabilized. In addition, since the growth curves of the WT and the mutant strains were different the values of activity were corrected for the number of CFU/mL present in the bacterial cultures. These calculations of the level of activity per cell revealed that a cell of the *∆pnpA* mutant produced from 2.18 to 3.16-fold- more bacteriocin than a cell of the WT depending on the growth phase (Fig. 1D). The most important cellular activity was registered in the *∆pnpA* mutant after 3 h of growth. Indeed, the cellular activity exhibited by the mutant strain at this time point was 2.67 × 10^−7^ ± 0.13 × 10^−7^ AU/CFU versus 0.84 × 10^−7^ ± 0.09 × 10^−7^ AU/CFU for the WT strain (P = 0.004). Furthermore, no significant variations were observed in the cellular activity of the WT strain at different time points (P > 0.05) (Fig. 1D).

**Figure 2:**
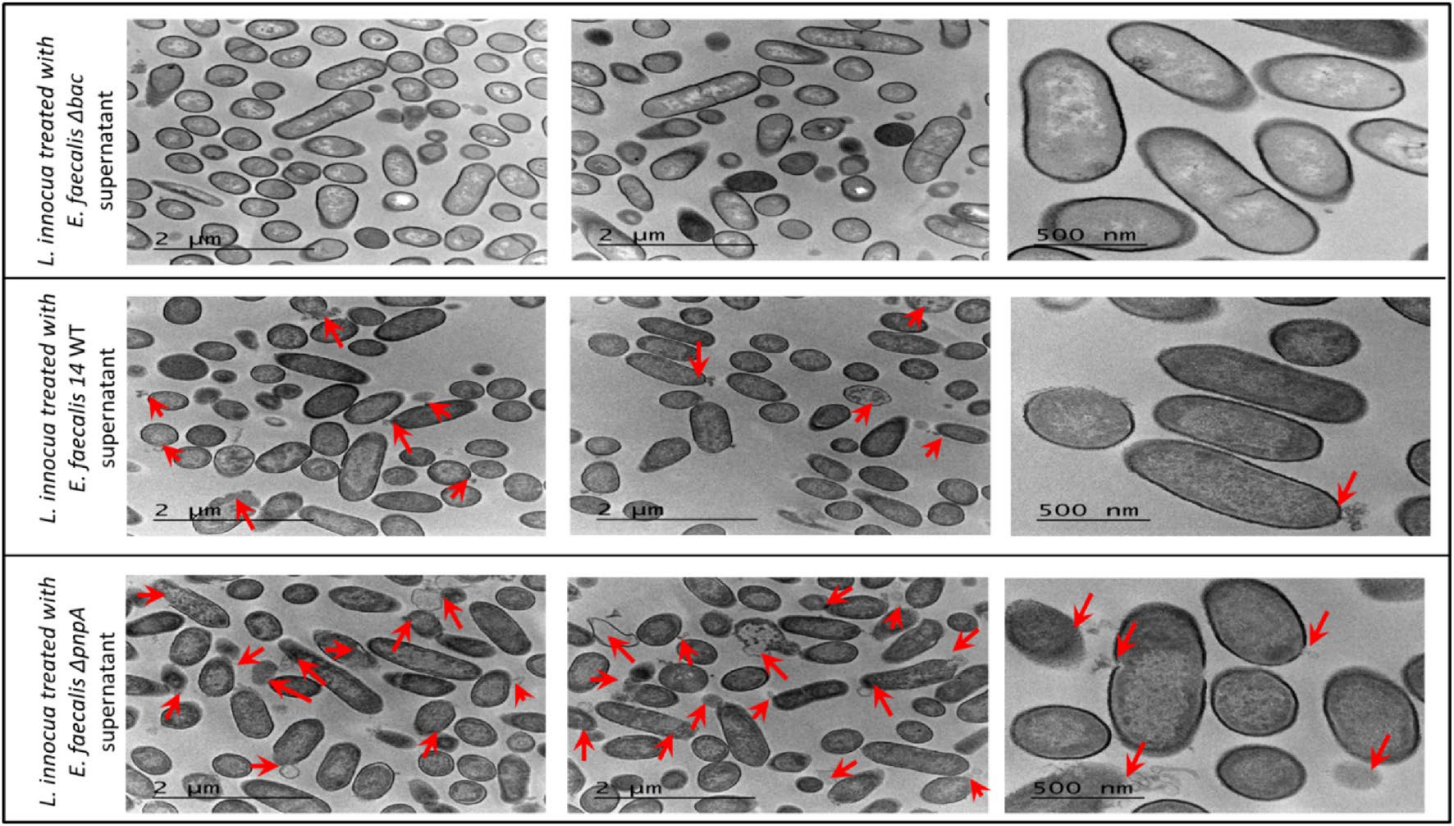
TEM micrographs of *Listeria innocua* ATCC33090 treated with CFS of *E. faecalis* WT*, ∆bac*, or *∆pnpA* mutant. Arrows indicate the main alterations in the ultrastructure of *L. innocua* ATCC33090. When *L. innocua* cells are treated with CFS containing EntDD14, alterations of the cell wall structure, condensation of ribosomes, separation of membrane layers and leakage of intracellular contents were observed.

The impact of EntDD14 present in the CFS of the WT and *∆pnpA* strains was examined on the ultrastructure of *L. innocua* ATCC 33090, through TEM observations. Thus, pellets of *L. innocua* ATCC 33090 were treated with CFS obtained from WT and *∆pnpA* strains and also CFS from the *∆bac* non-bacteriocin-producing strain used as a negative control. As shown on Fig. 2, micrographs from several samples revealed alterations and pores in the cell envelope structure of *L. innocua* ATCC33090. As expected, *L. innocua* ATCC 33090 treated with the CFS from the *∆bac* mutant strain did not reveal any abnormality in bacterial structure. However, the micrographs corresponding to *L. innocua* ATCC 33090 treated with the CFS from the* ∆pnpA* mutant revealed a profound impact and more morphological alterations than that of the WT (Fig. 2). Cellular alterations consist in disruption of the cell wall envelope, condensation of ribosomes, membrane layers separation and leakage of cytoplasmic contents, leading therefore to *L. innocua* cells-death.

### The *EntDD14* cluster is overexpressed in the* E. faecalis *Δ*pnp*A mutant

**Figure 3:**
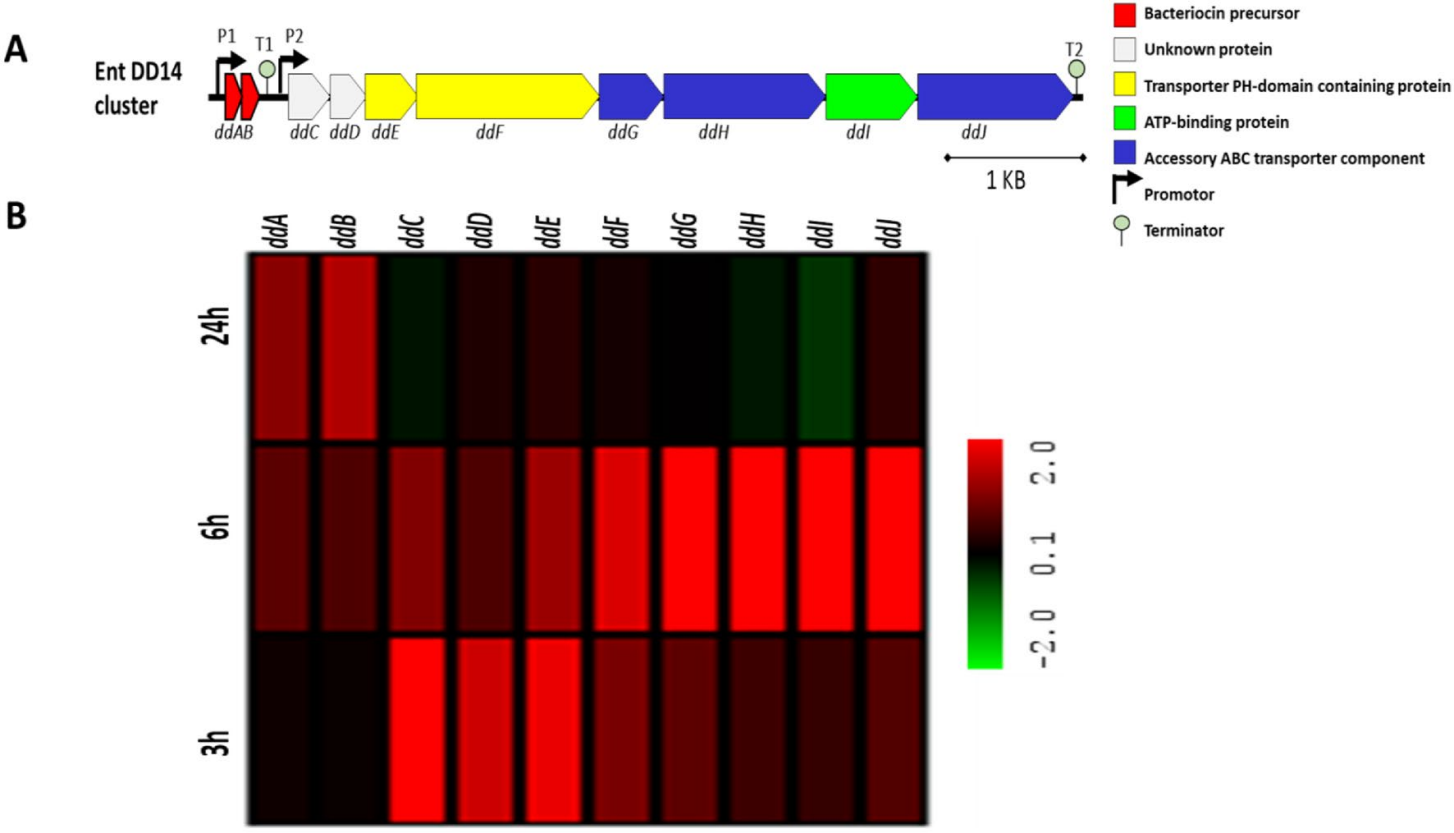
Gene expression profiles of DEGs involved in EntDD14 synthesis in the *E. faecalis ΔpnpA* mutant strain. Log2 FC of mean *ΔpnpA* mutant versus mean of WT were represented for cultures grown for 3, 6 and 24 h.

After 3 h of growth, the levels of *ddA* and *ddB* mRNAs detected in the ∆*pnpA* mutant did not significantly differ from the WT. However, when using probes matching the other genes from the *EntDD14* cluster, particularly the *ddCDEF* genes, higher levels of mRNAs were observed in the ∆*pnpA* mutant (Fig. 3B, Table S1). Next, after 6 h of growth, expression of all the genes present in the *EntDD14* cluster was detected at higher levels in the ∆*pnpA* mutant. The log2 ratio Δ*pnpA*/WT for *ddE* and *ddF* genes was 2.39 and 3.31, respectively. As for the *ddGHIJ* genes, the log2 ratio Δ*pnpA*/WT was 4.34 (*ddG*), 7.82 (*ddH*), 7.26 (*ddI*) and 5.29. After 24 h, only *ddA* and *ddB* genes were significantly expressed with a log2 ratio Δ*pnpA*/WT of 2.17 and 2.65, respectively.

**Figure 4:**
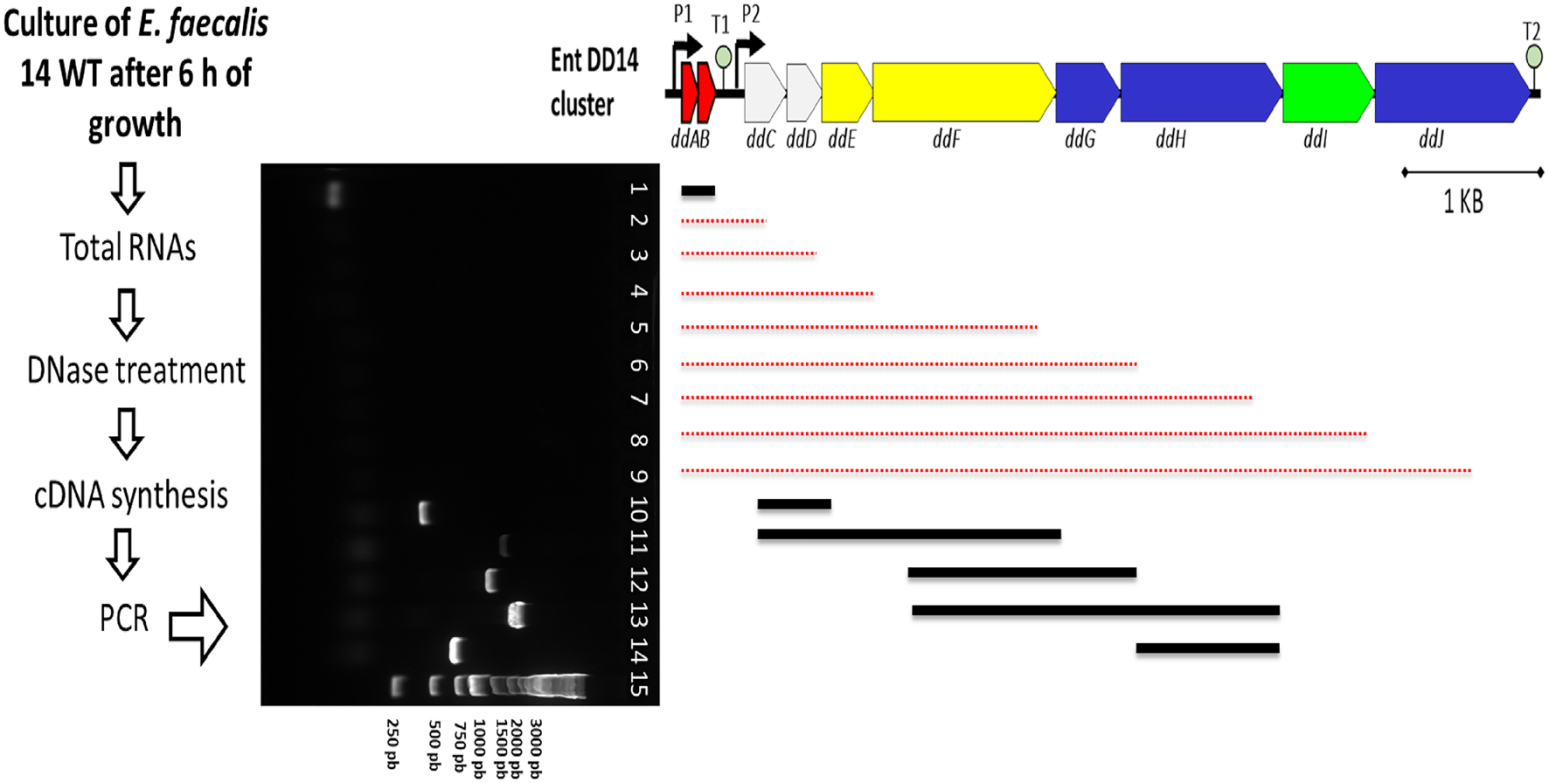
Electrophoresis gel of the PCR products using cDNAs obtained from total RNAs of *E. faecalis* 14 WT strain after 6 h of growth. The PCR products were generated using the following pairs of primers. Lane 1: ddA-F/ddb-R (87 pb), Lane 2: ddA-F/ddC-R, Lane 3: ddA-F/ddD-R, Lane 4: ddA-F/ddE-R, Lane 5: ddA-F/ddF-R, Lane 6: ddA-F/ddG-R, Lane 7: ddA-F/ddH-R, Lane 8: ddA-F/ddI-R, Lane 9: ddA-F/ddJ-R, Lane 10: ddC-F/ddD-R (429 pb), Lane 11: ddC-F/ddF-R (1420 pb), Lane 12: ddF-F/ddG-R (1161), Lane 13: ddF-F/ddH-R (1771), Lane 14: ddG-F/ddH-R (705) and Lane 15: molecular weight markers. The red dashed lines indicate the expected size of the target fragment if the fragment has been amplified.

Most of the genes present in the *EntDD14* cluster are adjacent or overlapping. However, an intergenic untranslated region of 216 nucleotides (nt) exists between the *ddb* and *ddc* genes. This fact, together with the detected differential pattern in RNA levels, indicated that probably *ddA* and *ddB* genes were co-transcribed, but the transcription of the other genes of the cluster was independent. To check this hypothesis, we performed PCR on cDNA obtained from total RNAs of WT after 6 h of growth (Fig. 4). The PCR amplification of these cDNAs using a set of primers (Table S2) revealed the presence of at least two transcription units. The first corresponds to mRNA co-transcribed from *ddA* and *ddB* genes and the second corresponds to mRNAs of the remaining *EntDD14* cluster (*ddCDEFGHIJ EntDD14*). It should be noted, that no transcript overlapping the two units was obtained, whatever the primer combination used (Fig. 4, red lines).

### In silico analyses of *EntDD14* cluster

The above results supported the existence of two transcripts within the *EntDD14* cluster. Therefore, an in-silico analysis was performed to identify transcriptional signals. Thus, two putative transcriptional promoters designated P1 and P2 were detected by inspecting the sequence of the *EntDD14* cluster. P1 and P2 promoters were located, respectively, 15 and 16 nt upstream of the translation start codon of *ddA* and *ddC*. The P1 promoter presented a − 35 (TTGAtA) and − 10 (aATAAT) regions, separated by 21 nt, and both boxes deviating only in one nucleotide from the canonical consensus sequences (TTGACA and TATAAT) for binding of the vegetative σ factor of the RNA polymerase. In the case of the P2 promoter, also a − 35 (TTGttA) and − 10 (TATAtT) regions with a spacing between the two boxes of 21 nt was detected.

**Figure 5:**
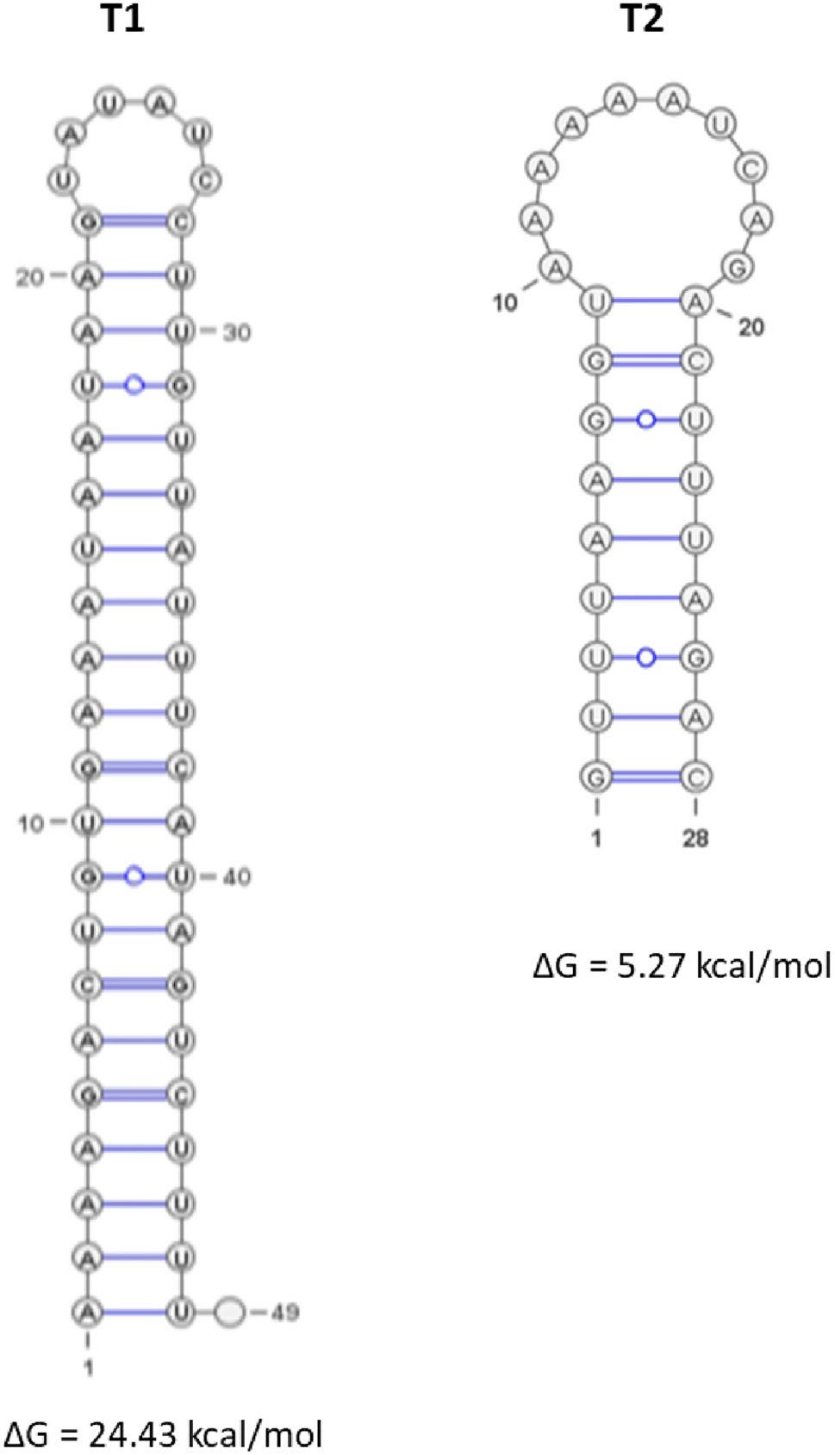
The T1 and T2 putative ρ-independent terminators. Their stem-loop structure and their ΔG were predicted with the RNA fold program.

Moreover, two putative ρ-independent transcriptional terminators designated T1 and T2 were identified (Fig. 5). The T1 is located between the 3′-end of *ddB* and the P2 promoter and their stem-loop structure predicted with the RNA Fold program has a Gibbs free energy (ΔG) with a value of − 24.43 kcal/mol. The T2 is located at 163 nt downstream of the 3-end of *ddG* and its predicted secondary structure has a ΔG = − 5.27 kcal/mol.

### Absence of PNPase activity results in a better *ddAB*, *ddH* and *ddJ* mRNA stability

**Figure 6:**
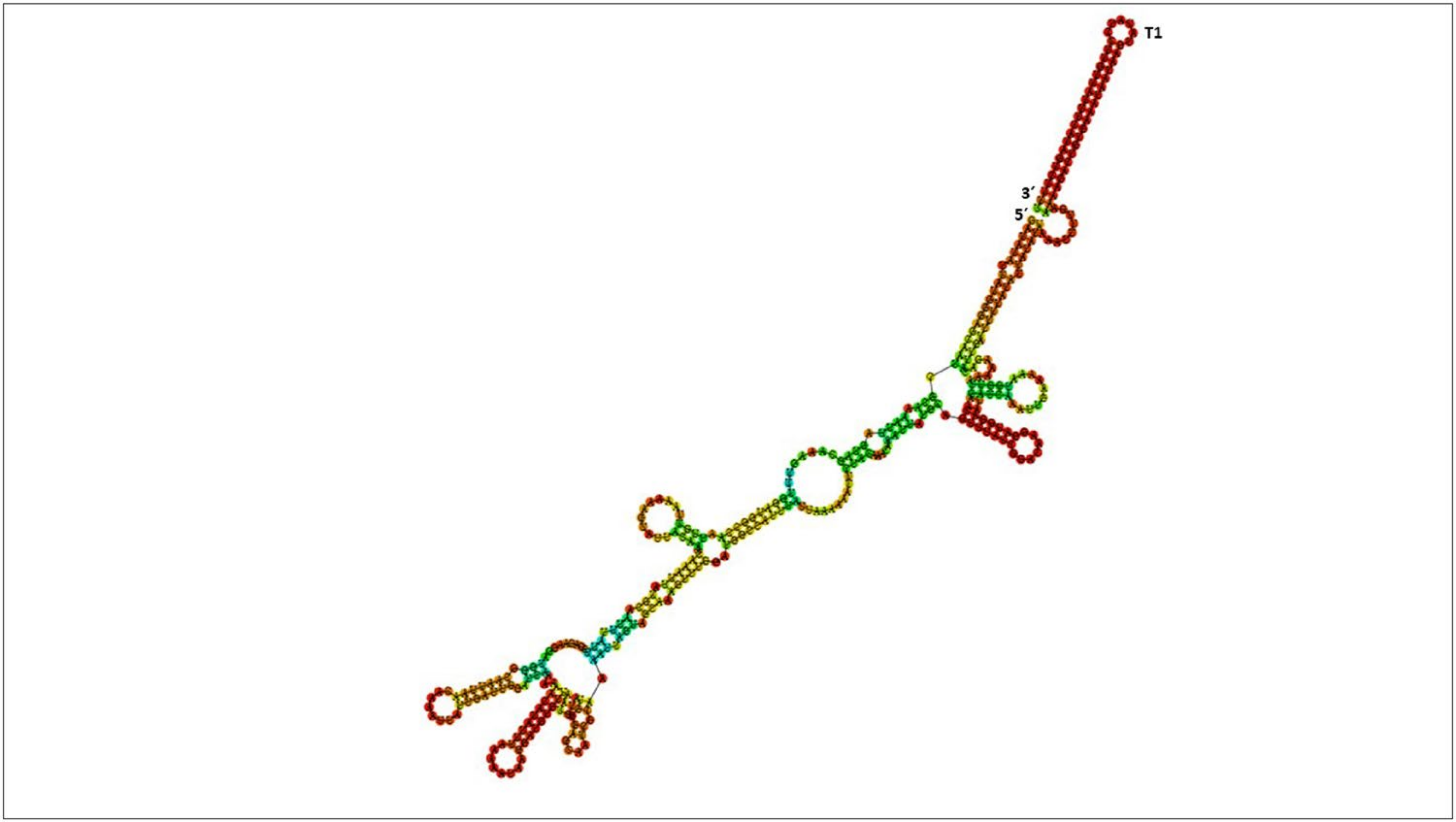
Prediction folding of the *ddAB* transcript. The prediction of the secondary structure of the mRNA was performed with the Vienna RNA program. The location of the 5′- and 3′-ends of the mRNA are indicated, as well as that of the T1 ρ-independent terminator.

**Figure 7:**
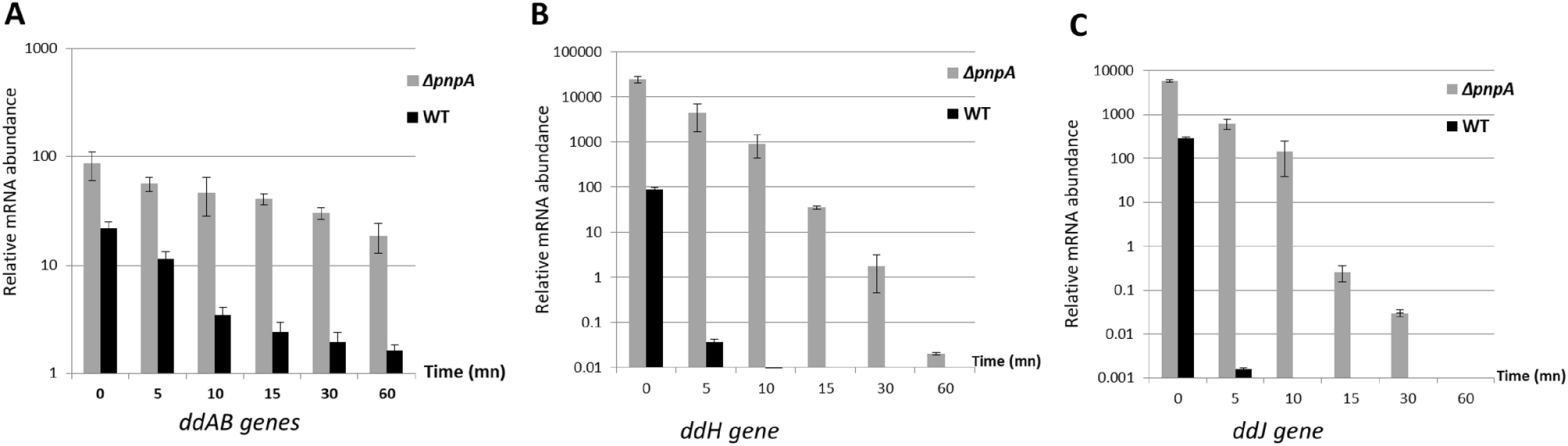
Fate of the *ddAB, ddH and ddJ* transcripts in WT and *∆pnpA* mutant at time points of 0 min, 5 min, 10 min, 15 min, 30 min and 60 min after stopping transcription. The vertical bars represent the standard deviations. The data are the means of three independent experiments.

Folding of the putative *ddAB* entire transcript revealed that in the presence of its whole RNA sequence, the transcriptional terminator T1 is expected to be formed (Fig. 6) and as a consequence to interfere with the PNPase 3′–5′ exonuclease activity. To study whether the fate of the *ddAB* transcript was affected by the absence of PNPase, we then examined the stability of *ddAB*, *ddH* and *ddJ* genes in the WT and *ΔpnpA* strains after 6 h of growth. The fate of the mRNA containing the *ddH* and *ddJ* genes were studied because *ddH* resulted to be the highest expressed in *∆pnpA* mutant after 6 h, also *ddJ* gene, which is the last gene of the second operon, and more exposed to PNPase activity. Transcription was blocked, upon addition of rifampicin to the cultures. Then, cultures samples were withdrawn at times points (0 min, 5 min, 10 min, 15 min, 30 min and 60 min) to determine the fate of the mRNAs, and their further degradation was impaired by immersion in liquid nitrogen (Fig. 6). At time point 0 min, the relative levels of the mRNAs of the *ddAB, ddH and ddJ* genes quantified by RT-qPCR were 3.92, 280.13 and 20.83-fold higher in the *ΔpnpA* mutant compared to the WT strain, leading to a log2 ratio Δ*pnpA*/WT of 1.97, 8.12 and 4.38 respectively. These log2 ratio Δ*pnpA*/WT were almost close to that obtained by microarray analysis after 6 h of growth with 1.70 (*ddAB*), 7.82 (*ddH*) and 5.29 (*ddJ*) (Fig. 3 and Table S1), indicating a good correlation between these two methods. Furthermore, our data showed that the level of *ddAB* mRNA decreased rapidly in the WT strain upon stopping transcription. The *ddAB* mRNA level decreased 13.55-fold within the interval 0–60 min in the WT strain, whereas in the *ΔpnpA*, it resulted to be more stable and decreased only by 4.64-fold (Fig. 7). The mRNA of *ddH* and *ddJ* were less stable in the WT strain than that of *ddAB*. A drastic degradation of these mRNAs was observed in the WT strain and any mRNA of *ddH* and *ddJ* was detected after 5 min following rifampicin addition. In contrast, in the *ΔpnpA* mutant*,* these mRNAs were detected even at 30 min for the *ddJ* gene and 60 min for the *ddH* gene (Fig. 7).

## Discussion

mRNA degradation is a key process for controlling gene expression in bacterial cells. It is carried out by the coordinated action of cellular endoribonucleases and exoribonucleases. Endoribonucleases cleave mRNAs at internal sites, whereas exoribonucleases do nucleolytic attacks from ends of the mRNA fragment, either 5′ or 3′ end based on their enzymatic specificity. It is noteworthy, that some bacteria such as *Mycobacterium tuberculosis* or *M. smegmatis* possess both 5′–3′ and 3′–5′ exoribonuclease activity, whereas other bacteria such as *E. coli* are only endowed with 3′–5′ exoribonuclease activity^27^. These ribonucleases usually require for their whole functionality the assistance of ancillary enzymes that covalently modify the 5′ or 3′ end of RNA or unwind base-paired regions^28^. One of the 3′–5′ exoribonucleases most studied is PNPase; an enzyme that form part of the degradosome, previously discovered in *E. coli*^29,30^. PNPase was found in different lineages of life such as in Bacteria, Archaea, Eukarya, organelles, animals and plants^31^, highlighting its conserved feature. Enzymatically, PNPase acts as a reversible enzyme that proceeds with 3′–5′ RNA degradation by using Mg^2+^ and inorganic phosphate (P_i_) or synthesize RNA by using any nucleoside diphosphate (rNDPs)^32,33^. PNPase is a non-essential enzyme that was proven to possess pleiotropic effects in different bacterial species and perturb the execution of genetic programs by causing drastic changes in global gene expression related to biofilm formation, growth at suboptimal temperatures, and virulence^34^. Recently, we reported the implication of PNPase in the global reorganization of the gene expression in *E. faecalis* 14, likely due to indirect transcriptional regulations^25^. Here, we show the involvement of PNPase in the expression of the *EntDD14* cluster coding for the synthesis of EntDD14 bacteriocin, which is naturally produced by *E. faecalis* 14^24^. The genetic determinants of the EntDD14 production, secretion and immunity constitute the *ddABCDEFGHIJ* cluster. The structural *ddA* and *ddB* genes encode EntDD14^26,35^. The functions of the *ddC* and *ddD* genes products are unknown and could serve in the bacteriocin self-immunity. The *ddE* and *ddF* encode an essential channel constituted by DdE and DdF, which serves to translocate EntDD14 outside of the cytoplasm^36,37^. Finally, *ddGHIJ* encode for an ABC transporter composed of the DdG, DdH, DdI, and DdJ proteins contributing to EntDD14 translocation and to resistance to this bacteriocin when it is externally supplied^36^. The synthesis of EntDD14 is more important in the Δ*pnp*A genetic background based on data generated by physiological, transcriptomic and mRNAs stability studies. The physiological study revealed the early stage of synthesis of EntDD14 in the growth kinetic and its level of production that was twofold higher in the Δ*pnp*A mutant (Fig. 1A–D). The TEM micrographs depicted on Fig. 2, indicate a severe impact of the CFS from the Δ*pnp*A mutant on the ultrastructure of the sensitive *L. innocua* ATCC33090, which is likely due to the abundance of EntDD14 in the CFS of Δ*pnp*A mutant. As a consequence of this impact, the cytoplasm content dissipates via the membrane, leading to cell death. Next, a transcriptomic microarray study exploiting mRNAs from both hosts, unraveled a positive deregulation of the *EntDD14* cluster expression in the Δ*pnpA* mutant (Fig. 3). Taken together, these microbiological and transcriptomic analyses indicate a potential role of PNPase in the expression of *ddA* and *ddB* genes and synthesis of EntDD14. To strengthen these data, we analyzed in-silico the *EntDD14* cluster sequence and determined experimentally its mRNAs decay. The in-silico analysis enabled to locate two putative ρ-independent transcriptional terminators, designated T1^26^ and T2, with a clear difference in their ΔG (Fig. 5). It should be stated, that T1 should be a strong transcriptional terminator, unlike T2, acting also as a strong barrier for the mRNA 3′–5′ degradation involving the PNPase in the WT, contrarily to T2. In the Δ*pnp*A mutant, the lack of PNPase should not have a drastic effect in the half-life of the *ddAB* transcript, correlating with the 2.6-fold-increase detected after 24 h of growth. By contrast, the *ddCDEFGHIJ* transcript, with such unstable structure of its T2 terminator, should be a perfect target for the PNPase activity and in its absence, the stability of the transcript should be drastically increased as observed by transcriptomic method for some genes like *ddH* or *ddJ*, which had reached respectively a 7.82-fold and 5.29-fold increase after 6 h of growth. To confirm these hypotheses, we established as indicated on Fig. 4, the cotranscription of *ddA* and *ddB* genes on one hand, and that of *dCDEFGHIJ* genes, on the other hand. The first transcriptional unit (*ddAB*) is involved in the synthesis of the two-peptides DdA and DdB of EntDD14, while the second (*dCDEFGHIJ*) is implicated in the immunity and translocation of EntDD14 out of the cell-membrane. The level of EntDD14 in the Δ*pnp*A mutant could be a direct consequence of the *pnpA* deletion on the in vivo degradation activity or indirect changes in mRNA concentration provoked by this mutation, as reported elsewhere^38^. Notably, mRNA decay was proven to be governed by diverse factors like RNA sequence and structure, translating ribosomes, and bound sRNAs or proteins^28^. One of the first steps to understand the mechanism of action of an RNA is to determine its secondary structure^39^. Related to that, the secondary structure of *ddAB* mRNA was predicted (Fig. 6), its stability was determined, and compared to those of *ddI* and *ddJ,* whose relative levels of the mRNAs were almost similar after 6 h of growth (Fig. 3 and Table S1). Moreover, *ddAB* mRNA decayed rapidly in the WT, but was more stable in the *ΔpnpA* mutant (Fig. 7). Regarding to the second transcriptional unit, we observed a drastic decay of the *ddH* and *ddJ* mRNAs in the WT strain, only 5 min after transcription inhibition. Moreover, a pronounced increase of the *ddH* and *ddJ* mRNAs stability was observed in the *ΔpnpA* background, the transcript still being detected 30 min (*ddJ* gene), and 60 min (*ddH* gene) after rifampicin addition (Fig. 7), and that showing the role of PNPase in this regulation.

To sum up, bacteriocins are produced at low yields by a single bacterial strain, and their purification in such conditions are cost-effective, tedious and time consuming. To scale-up the production of bacteriocins, different studies suggested the use of a concomitant culture of a bacteriocin producing strain with a target sensitive strain, or regulation through the Quorum-Sensing mechanism^40^. Other studies suggested their heterologous production in bacteria and yeasts^16,19,41^. Here we show that increase in bacteriocin production can be achieved by elimination of PNPase activity in the producing strain. As far as we know, this is the first report highlighting the implication of PNPase in controlling gene expression and mRNA stability of a bacteriocin, namely EntDD14.

## Materials and methods

### Bacterial strains and growth conditions

In this study the EntDD14-producing *E. faecalis* 14 strain (designated WT)^24^, as well as the previously constructed isogenic strains: the *∆pnpA* mutant lacking the PNPase activity^25^ was used and the *∆bac* mutant deficient in the EntDD14 *ddA* and *ddB* structural genes^25^. In addition, *Listeria innocua* ATCC 33090 was used as a sensitive strain to test EntDD14^42^. These two mutant strains were previously constructed by a double homologous recombination using the pLT06 vector^43^. The *E. faecalis* strains were grown in GM17 (M17 medium containing glucose at 0.5% (w/v)) and *L. innocua* ATCC33090 was grown in BHI. All strains were incubated at 37 °C without shaking. GM17 Agar plates were used to determine the number of viable cells (CFU/mL). The growth kinetics were carried out using a spectrophotometer (Aquoalabo, France) set at 600 nm.

The same culture volume of the wild-type and *ΔpnpA* mutant was used, for all the experiments detailed below, because at each sampling time (3 h, 6 h, 9 h and 14 h) non-significant differences (P > 0.05) were observed between both strain for the values of colony forming units (CFU) per mL (data not shown), as previously reported^25^. Thus, for example the mean values of viable cells after 6 h were 1.26 × 10^9^ ± 0.48 × 10^9^ CFU/mL for the *ΔpnpA* mutant and 1.40 × 10^9^ ± 0.18 × 10^9^ CFU/mL for the WT strain (P > 0.05).

### Antibacterial activity

The antibacterial activity of the WT and *∆pnpA* strains against *Listeria innocua* ATCC 33090 was assessed by using the well-diffusion and the spot-on-lawn methods^44^. Briefly, BHI plates containing 1% agar were inoculated with *L. innocua* ATCC 33090 and allowed to dry at room temperature. For the spot-on-lawn method, 4 μL of bacterial culture were spotted onto the plate. For the well diffusion method, cell-free supernatant (CFS) of the WT and *∆pnpA* strains were obtained by centrifugation (10,000×*g*, 20 min, 4 °C). The resulting CFS was serially diluted two-fold with filter-sterilized 20 mM phosphate buffer (pH 6·5). Afterwards, 50 µL of the samples were poured into the wells of 6 mm diameter previously made in the BHI-agar plates. The plates were first incubated at 4 °C for 1 h and then overnight at 37 °C. The radius of the halo was measured from the edge of the well/spot to the edge of the halo. A clear halo of at least 2 mm in diameter was recorded as positive. Antibacterial activity was quantified using the Arbitrary Units per milliliter (AU/mL) according to the following formula: 2^n^ × (1000 μL/deposited volume 50 µL), with n corresponding to the highest number of dilution at which growth inhibition of the sensitive strain was observed^45^. To determine the cellular activity (CA) corresponding to the production of bacteriocin by individual cell, the following formula was used: CA (AU/CFU) = Antibacterial activity quantified in (AU/mL)/number of CFU quantified in (CFU/mL). For the specific activity (SA) of the culture supernatants of the WT and *∆pnpA* strains the following formula was used: SA (AU/mg) = Antibacterial activity quantified in (AU/mL)/Total protein concentration of the cell-free supernatants (mg/mL).

### Transmission electron microscopy

The transmission electron microscopy (TEM) analysis was performed using the same method previously described^26^. Briefly, cultures of *L. innocua* ATCC33090 grown in BHI at 37 °C for 6 h were harvested by centrifugation (10,000×*g*, 10 min, 4 °C). Then, pellets were resuspended in the cell-free supernatant of either the WT or *∆pnpA* strains and incubated overnight at room temperature. A pellet of *L. innocua* ATCC33090 treated in identical conditions with a CFS of *∆bac* strain was used as a negative control, since this null-mutant does not produce EntDD14. Pellets obtained after different treatments were fixed with 2.5% (v/v) glutaraldehyde solution and 0.1 M (v/v) of cacodylate buffer (pH 7.4), and prepared as a Formvar film on a 300 square mesh, nickel grid (EMS FF300-Ni). TEM images were obtained with a JEOL JEM 2100FX TEM instrument (Jeol, Tokyo, Japan), equipped with a GATAN CCD Orius 200D camera (Gatan, Pleasanton, CA, USA) and used at an acceleration voltage of 200 kV.

### RNA isolation and microarrays analysis

*E. faecalis* 14 WT and ∆*pnpA* strains were grown in GM17 medium. After 3 h, 6 h and 24 h of growth, bacterial cells were harvested by centrifugation (10,000×*g*, 10 min, 4 °C) and total RNAs were extracted using the NucleoSpin™ RNA Plus columns (Macherey-Nagel, Hoerdt, France). The Agilent 2100 Bioanalyzer (Agilent Technologies, France) was used to determine the quality and quantity of RNA samples and a RIN of 8 (minimal RNA integrity number) was required for all samples. A custom *E. faecalis* 14 oligo-based DNA microarray (8 × 15 K) Agilent G2509F was used to study the gene expression using the method previously reported^46^. Genes of EntDD14 cluster *ddABCDEFGHIJ* were selected for this study, and the log2 ratio from individual *ΔpnpA* samples and the mean of WT samples were calculated. The corresponding probes designed for these genes were previously reported^26^. These microarray data were submitted to the NCBI GEO with the accession number GSE180397.

### Stability of mRNAs of *ddAB*, *ddH *and *ddJ* genes

Three distinct cultures of 50 mL of WT and ∆*pnpA* strains were grown in GM17 at 37 °C. After 6 h of growth, rifampicin at 300 µg/mL was added to the cultures. Of note, the first sample was taken prior addition of rifampicin (initial mRNA t0) and was used as control, after which rifampicin was added, then 10 mL of cultures were withdrawn after 5 min, 10 min, 15 min, 30 min and 60 min and transferred immediately into liquid nitrogen. Three independent samples of total RNAs were isolated from each strain by using NucleoSpin™ RNA Plus columns (Macherey-Nagel, Hoerdt, France). The quality and quantity of RNA samples were determined by Agilent TapeStation 4150 (Agilent Technologies, France), and a minimal RIN of 8.5 was retained for all samples. A quantity of 1 µg of RNA of each sample was firstly treated with DNase I (Thermo Fisher scientific), and then used as substrate for cDNA synthesis with the RevertAid RT Reverse Transcription Kit (Thermo Fisher scientific). For quantitative PCR (RT-qPCR). Specific primers for *ddAB*, *ddH* and *ddJ* genes (ddA-F/ddB-R, ddH-F/ddH-R, ddJ-F/ddJ-R respectively; Table S2) were designed using the WT genome sequence and Primer3 software (https://bioinfo.ut.ee/primer3-0.4.0/). PCR was performed using cDNA dilutions of 1:100 and DNA polymerase Brilliant III SYBR Green QPCR Master Mix (Agilent Technologies). Detection of the threshold value, and the real-time analysis were performed three times for each cDNA sample using the CFX Connect Real-Time PCR Detection System thermocycler (BIO-RAD). Relative *ddAB, ddH* and *ddJ* mRNA levels in each sample were calculated using comparative cycle time data^47^.

### Detection of different mRNA transcripts of the EntDD14 cluster and prediction of *ddA* and *ddB* mRNAs secondary structures

cDNAs obtained from total RNAs of the WT culture after 6 h of growth were used as a template for PCR using several pairs of primers specific to the *ddABCDEFGHIJ* genes (Table S2). The detection of a fragment of the expected size on an agarose gel, indicates the presence of a transcript linking the two targeted genes. Predictions of the secondary structures of the transcript of the *EntDD14* cluster and of the transcriptional terminators were obtained by using the RNAfold web server (The ViennaRNA Web Services, http://rna.tbi.univie.ac.at/) and edited with VARNA 3.9 software^48^.

### Statistics

All the results of this study are obtained from at least three independent experiments. They are expressed as the mean with the standard deviation. *P* values < 0.05 were considered to be significant using the student test.

### Supplementary Information

Supplementary Figures.

Supplementary Tables.

## Data Availability

The accession number of the *E. faecalis* 14 chromosome is CP021161.1 (https://www.ncbi.nlm.nih.gov/nuccore/CP021161.1?report=genbank). The microarray data from this study have been submitted to the NCBI GEO with the accession number GSE180397 (https://www.ncbi.nlm.nih.gov/geo/query/acc.cgi?acc=GSE180397).
