# Supplementary figures and images for: Lack of PNPase activity in Enterococcus faecalis 14 increases the stability of EntDD14 bacteriocin transcripts
Source: Sci Rep. 2023 Dec 18;13:22870. doi: 10.1038/s41598-023-48619-y (PMC10739964; doi:10.1038/s41598-023-48619-y)

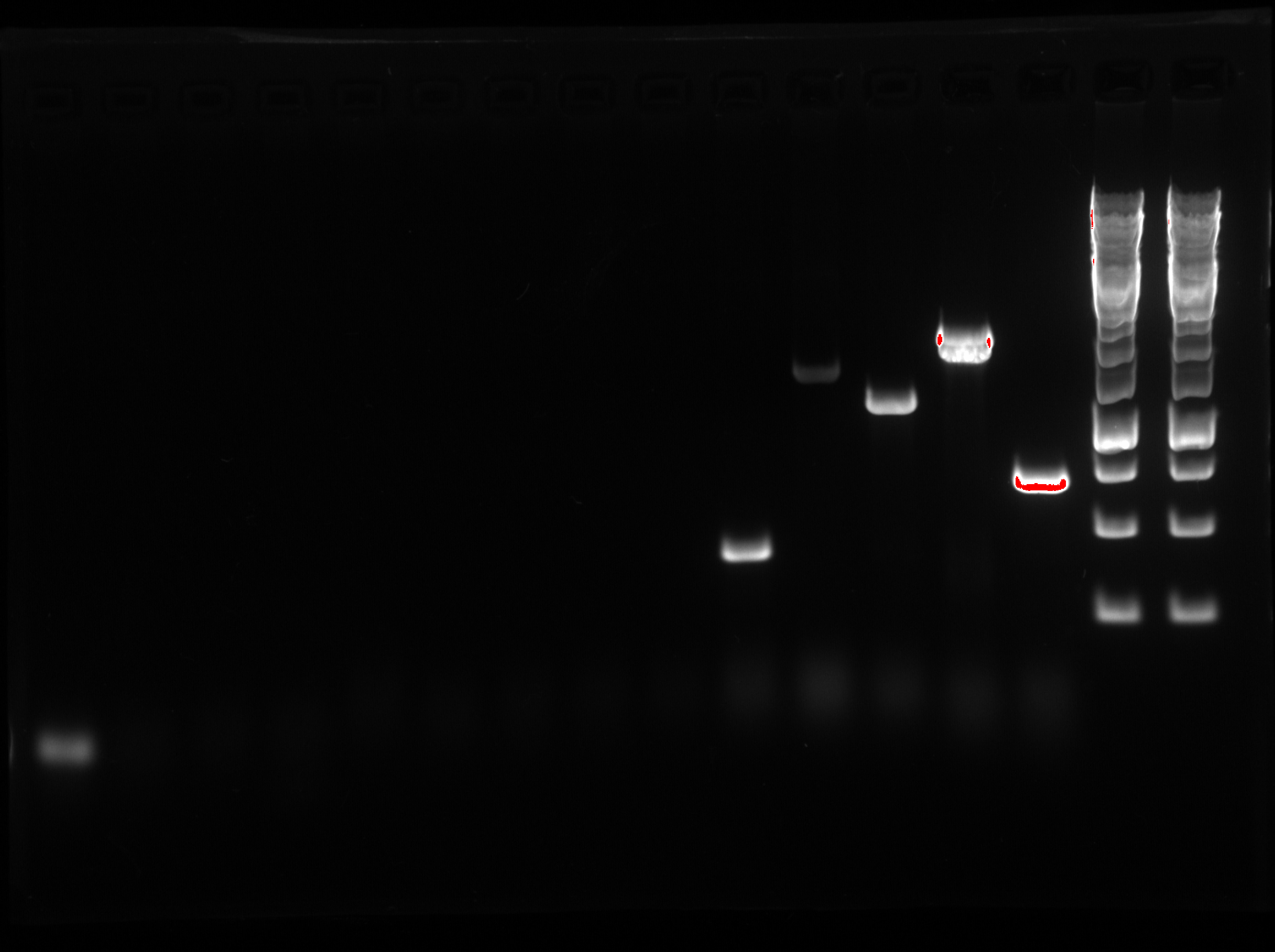

Supplement: Supplementary file 1 — Supplementary Figures. [file 41598_2023_48619_MOESM1_ESM.tif]
