# Supplementary material for: Lack of PNPase activity in Enterococcus faecalis 14 increases the stability of EntDD14 bacteriocin transcripts
Source: Sci Rep. 2023 Dec 18;13:22870. doi: 10.1038/s41598-023-48619-y (PMC10739964; doi:10.1038/s41598-023-48619-y)
Supplement: Supplementary file 2 — Supplementary Tables. [file 41598_2023_48619_MOESM2_ESM.docx]

**Table S1.** Gene expression profiles in *Enterococcus faecalis* *ΔpnpA* mutant strain at 3h, 6h and 24h of growth. Log2 of fold change for each individual *ΔpnpA* (1-3) for up- and down-regulated genes, log2 ratio threshold >1 and <-1 respectively.

| **Gene name** | **ID** | **P/WT 3h** | **P/WT 6h** | **P/WT 24h** | **Functional annotation** |
| --- | --- | --- | --- | --- | --- |
| ***ddA*** | CUST_250_PI442896112 | 1.16032436 | 1.70863 | **2.179934** | Laederless two-peptide bacteriocin |
| ***ddB*** | CUST_251_PI442896112 | 1.13167855 | 1.59923 | **2.6590171** | Laederless two-peptide bacteriocin |
| ***ddC*** | CUST_2_PI443061160 | **4.4480869** | **2.08155** | 0.9661017 | hypothetical protein |
| ***ddD*** | CUST_3_PI443061160 | **3.0824688** | **1.59901** | 1.2596149 | hypothetical protein |
| ***ddE*** | CUST_4_PI443061160 | **3.62236167** | **2.39698** | 1.3228553 | PH domain-containing protein YdbT- bacteriocin transporter |
| ***ddF*** | CUST_5_PI443061160 | **2.00400751** | **3.31504** | 1.1988066 | PH domain-containing protein - bacteriocin transporter |
| ***ddG*** | CUST_6_PI443061160 | 1.72669029 | **4.34193** | 1.0837606 | Accessory ABC transporter component |
| ***ddH*** | CUST_7_PI443061160 | 1.46533908 | **7.82981** | 0.9474638 | putative efflux Resistance-nodulation-division (RND) transporter periplasmic adaptor subunit |
| ***ddI*** | CUST_8_PI443061160 | 1.41089102 | **7.26768** | 0.8073924 | ATP-binding protein of the ATPase family |
| ***ddJ*** | CUST_9_PI443061160 | 1.63748068 | **5.29895** | 1.35961 | putative macrolide export ATP-binding/permease protein (MacB) |

**Table S2.** Oligonucleotides sequences used in this study

| Name | Sequence 5'🡪3' |
| --- | --- |
| ddA-F | GGAGAAGGATGGGCAATTAAC |
| ddb-R | CGATTGCTCCCATACATTAACA |
| ddC-F | ATCGAAAATGTGTAAAAAGGGATA |
| ddC-R | TCTTGTGAATACCTTGCTTTACCT |
| ddD-F | TTATAGGATTGATGTTTGGATTCTTT |
| ddD-R | TTTTGTCTCTCTTTGTTTATCCTATCC |
| ddE-F | ACAAGAACATATACATTTGTGAAGGA |
| ddE-R | AACATATTCTGTTTCAATTACCGTGT |
| ddF-F | AGGAAAATGTTGATTTGGTGTTT |
| ddF-R | TCCAATGAAGATAACAAGACAAAAA |
| ddG-F | TTATCAAAAACTTGGCAATGATG |
| ddG-R | TGACTATCAACAAATTTAAAACAGCA |
| ddH-F | TGGTCAAGAAATCAATGAAAATG |
| ddH-R | CTAGAGATTGGGTTTGTTCTTCC |
| ddI-F | GGGATTTATCGATCGTAAGTTTG |
| ddI-R | TTTTAGAAAGAATGTCATCTGCTGT |
| ddJ-F | AGAAGGAGTTAAACCCGATAAGG |
| ddJ-R | TCATATTCTCCCAGATGTCTCAA |
